# Supplementary material for: Suitability of GnRH Receptors for Targeted Photodynamic Therapy in Head and Neck Cancers
Source: Int J Mol Sci. 2019 Oct 11;20(20):5027. doi: 10.3390/ijms20205027 (PMC6829278; doi:10.3390/ijms20205027)
Supplement: Supplementary file 1 [file ijms-20-05027-s001.docx]

**Supplementary materials**

Suitability of GnRH receptors for targeted photodynamic therapy in head and neck cancers

Lilla Pethő^1^, József Murányi^2^, Kinga Pénzes^2^, Bianka Gurbi^2^, Diána Brauswetter^2^, Gábor Halmos^3^, Gabriella Csík^4^, Gábor Mező^1,5*^

^1^ MTA-ELTE Research Group of Peptide Chemistry, Budapest, Hungary

^2^ MTA-SE Pathobiochemistry Research Group, Budapest, Hungary

^3^ Department of Biopharmacy, Faculty of Pharmacy, University of Debrecen, Debrecen, Hungary

^4^ SE Department of Biophysics and Radiation Biology, Budapest, Hungary

^5^ ELTE Eötvös Loránd University, Faculty of Science, Institute of Chemistry, Department of Organic Chemistry, Budapest, Hungary

***** Correspondence: gmezo@elte.hu

**Analytical data**

**Table S1** Analytical characteristics of the peptide-PpIX conjugates.

| **Code** | **Conjugate** | ***R_t_* (min)*^a^*** | ***MW_calc._ / MW_meas._^b^*** |
| --- | --- | --- | --- |
| 5 | GnRH-I[^6^*D*-Lys(PpIX)] | 24.2 | 1796.9 / 1797.4 |
| 6 | GnRH-I[^4^Lys(Bu), ^6^*D*-Lys(PpIX)] | 24.7 | 1908.0 / 1908.5 |
| 7 | GnRH-II[^4^Lys(Bu), ^6^*D*-Lys(PpIX)] | 24.9 | 1962.0 / 1962.7 |
| 8 | GnRH-III[^4^Lys(Bu), ^6^Asp(OMe), ^8^Lys(PpIX)] | 25.1 | 1927.9 / 1928.6 |

*^a^* Knauer RP-HPLC; Nucleosil C18 column (5 μm, 100 Å; 250×4.6 mm) gradient: 0 min 2% B, 5 min 2% B, 30 min 90% B; eluents: 0.1% TFA in water (A) and 0.1% TFA in acetonitrile (B); flow rate: 1 mL/min, detection: λ = 220 nm.

*^b^* Bruker Daltonics Esquire 3000+ ESI-MS, 10 μL/min flow rate, positive ion mode in the *m/z* 50–2000 range.


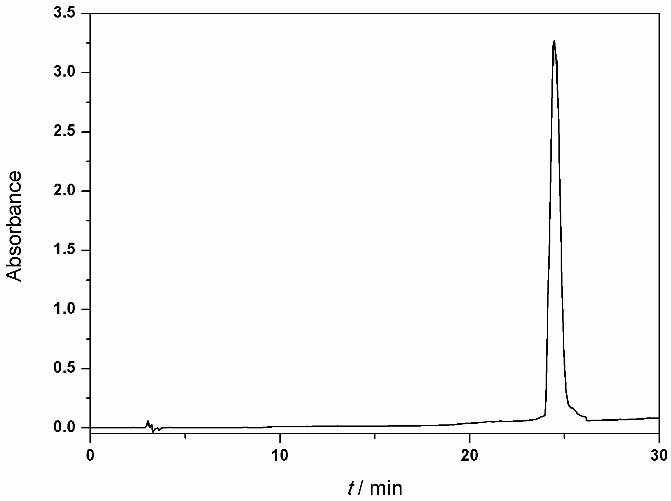

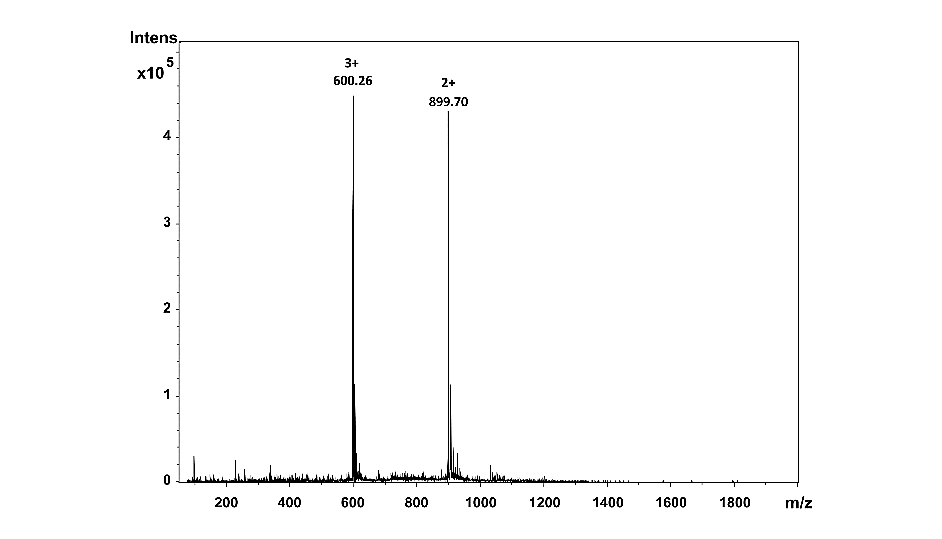


**Figure S1.** Analytical RP-HPLC chromatogram and ESI-MS spectrum of **5**


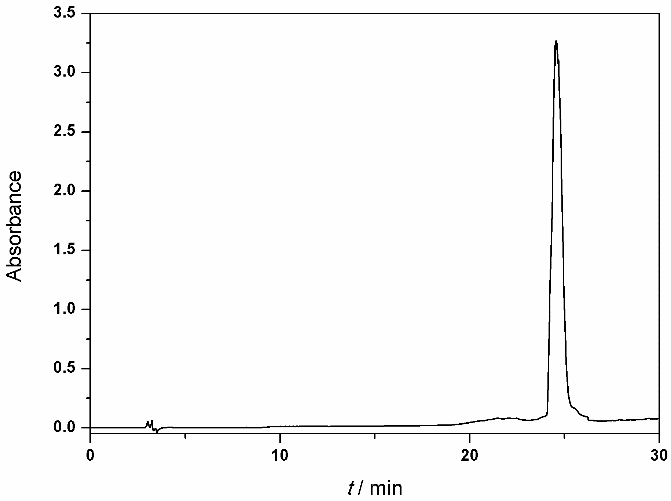

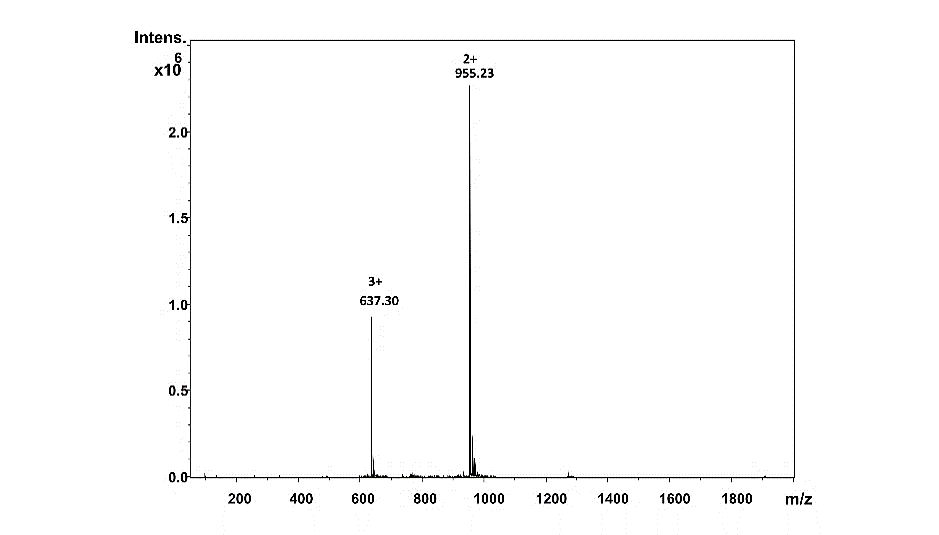


**Figure S2.** Analytical RP-HPLC chromatogram and ESI-MS spectrum of **6**


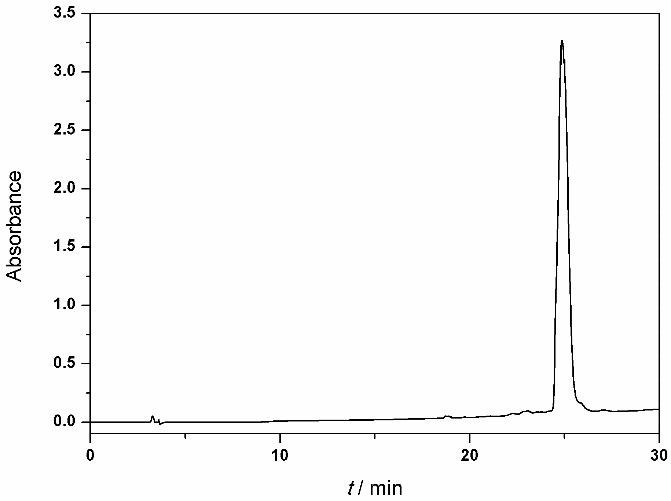

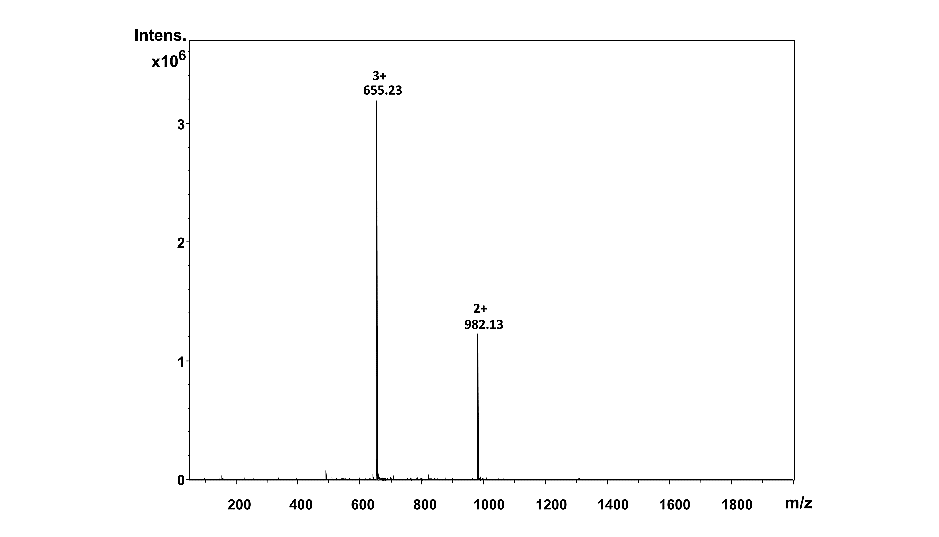


**Figure S3.** Analytical RP-HPLC chromatogram and ESI-MS spectrum of **7**


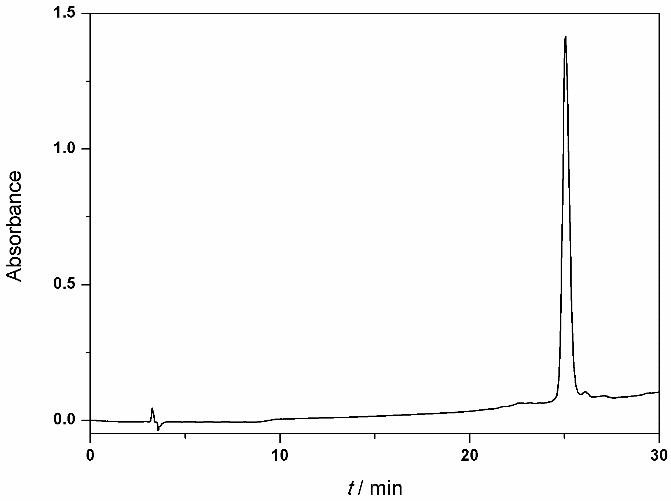

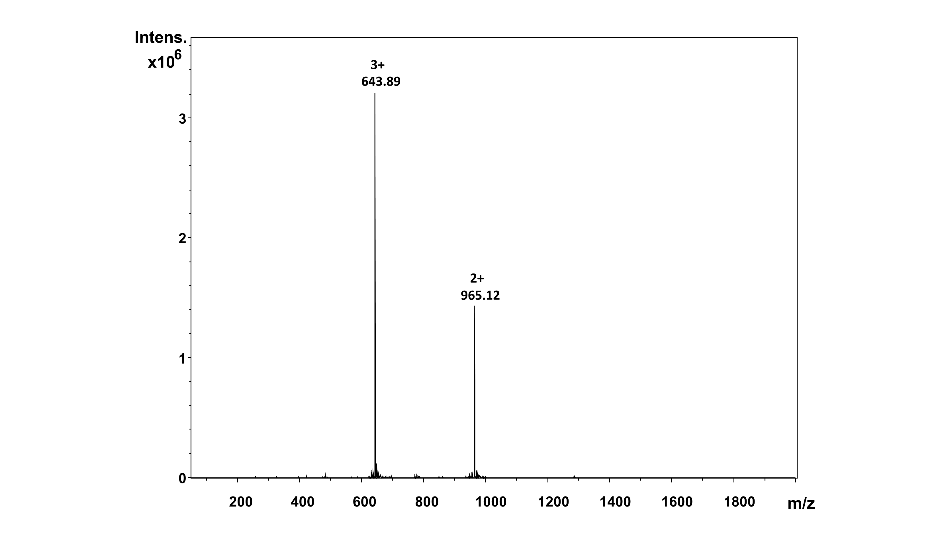


**Figure S4.** Analytical RP-HPLC chromatogram and ESI-MS spectrum of **8**

**UV-Vis spectroscopy**


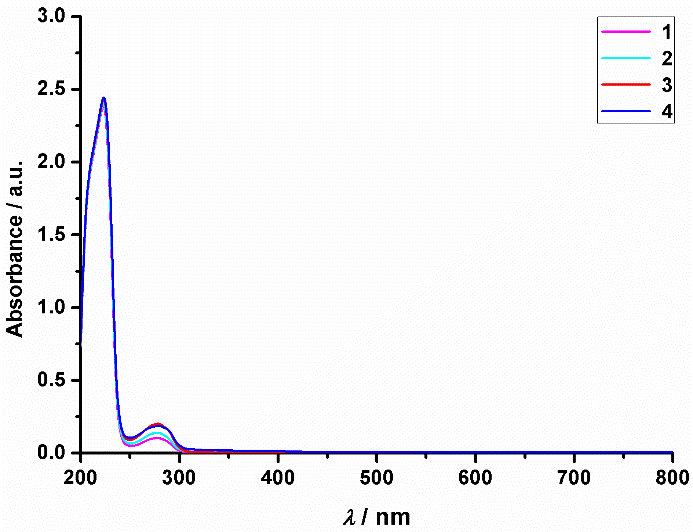


**Figure S5.** UV-Vis absorbance of compounds **1**-**4**

***In vitro* cell viability inhibition assay**


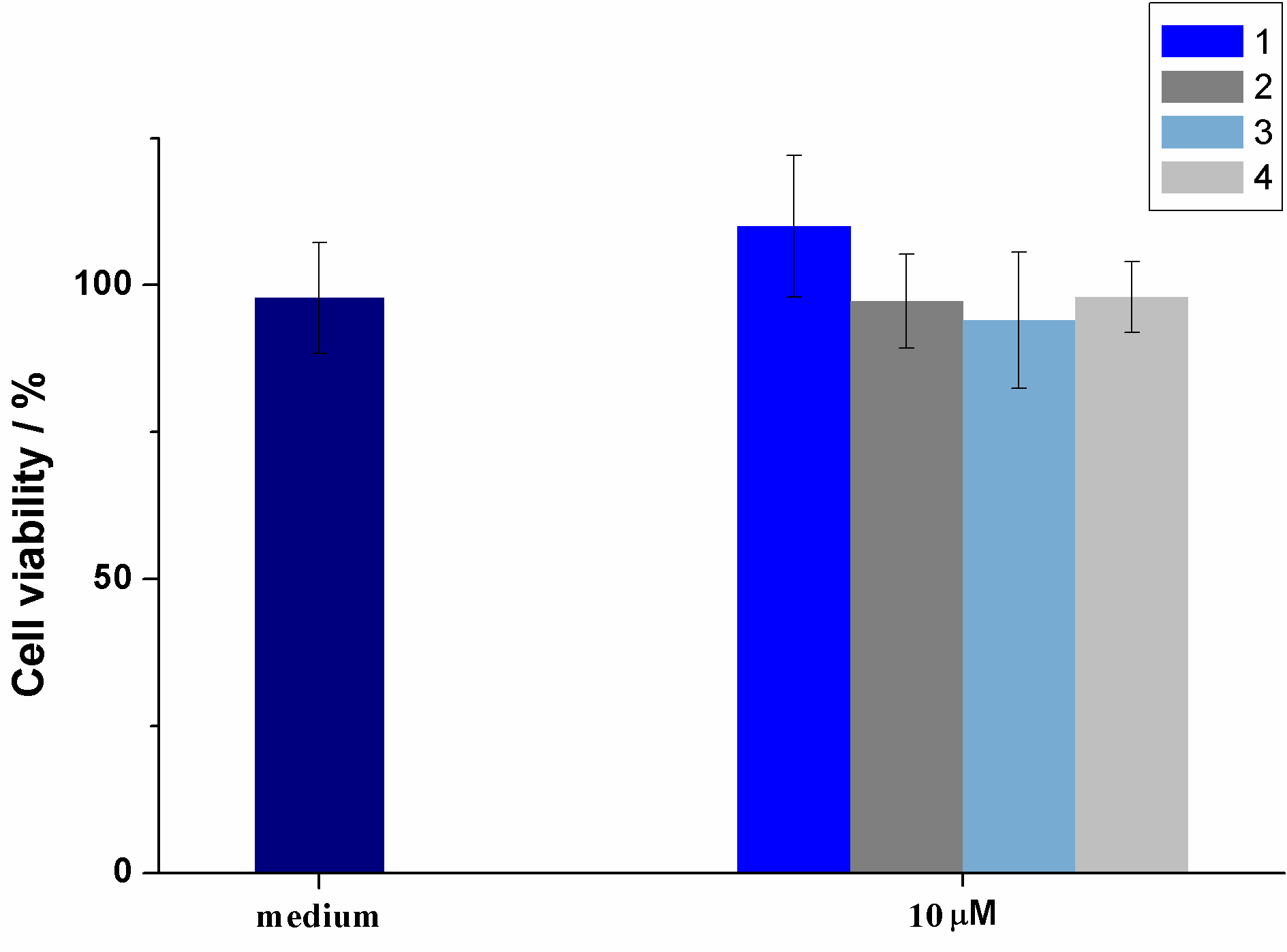


**Figure S6.** *In vitro* cell viability inhibition of compounds **1**-**4** and cell culture medium. Cell viability values are present in % of DMSO control.
